# A novel small-molecule compound targeting CD147 inhibits the motility and invasion of hepatocellular carcinoma cells

**DOI:** 10.18632/oncotarget.6990

**Published:** 2016-01-23

**Authors:** Zhi-guang Fu, Li Wang, Hong-yong Cui, Jian-long Peng, Shi-jie Wang, Jie-jie Geng, Ji-de Liu, Fei Feng, Fei Song, Ling Li, Ping Zhu, Jian-li Jiang, Zhi-nan Chen

**Affiliations:** ^1^ Cell Engineering Research Center & Department of Cell Biology, State Key Laboratory of Cancer Biology, National Key Discipline of Cell Biology, Fourth Military Medical University, Xi'an, P.R. China; ^2^ State Key Laboratory of Cancer Biology, Department of Pharmacogenomics, School of Pharmacy, Fourth Military Medical University, Xi'an, P.R. China; ^3^ Drug Discovery and Design Center, State Key Laboratory of Drug Research, Shanghai Institute of Materia Medica, Chinese Academy of Sciences, Shanghai, P.R. China; ^4^ Department of Clinical Immunology, PLA Specialized Research Institute of Rheumatology & Immunology, Xijing Hospital, Fourth Military Medical University, Xi'an, P.R. China

**Keywords:** CD147, small molecule inhibitor, hepatocellular carcinoma cells, metastasis, dimerization

## Abstract

CD147, a type I transmembrane glycoprotein, is highly expressed in various cancer types and plays important roles in tumor progression, especially by promoting the motility and invasion of hepatocellular carcinoma (HCC) cells. These crucial roles make CD147 an attractive target for therapeutic intervention in HCC, but no small-molecule inhibitors of CD147 have been developed to date. To identify a candidate inhibitor, we used a pharmacophore model derived from the structure of CD147 to virtually screen over 300,000 compounds. The 100 highest-ranked compounds were subjected to biological assays, and the most potent one, dubbed AC-73 (ID number: AN-465/42834501), was studied further. We confirmed that AC-73 targeted CD147 and further demonstrated it can specifically disrupt CD147 dimerization. Moreover, molecular docking and mutagenesis experiments showed that the possible binding sites of AC-73 on CD147 included Glu64 and Glu73 in the N-terminal IgC2 domain, which two residues are located in the dimer interface of CD147. Functional assays revealed that AC-73 inhibited the motility and invasion of typical HCC cells, but not HCC cells that lacked the CD147 gene, demonstrating on-target action. Further, AC-73 reduced HCC metastasis by suppressing matrix metalloproteinase (MMP)-2 via down-regulation of the CD147/ERK1/2/signal transducer and activator of transcription 3 (STAT3) signaling pathway. Finally, AC-73 attenuated progression in an orthotopic nude mouse model of liver metastasis, suggesting that AC-73 or its derivatives have potential for use in HCC intervention. We conclude that the novel small-molecule inhibitor AC-73 inhibits HCC mobility and invasion, probably by disrupting CD147 dimerization and thereby mainly suppressing the CD147/ERK1/2/STAT3/MMP-2 pathways, which are crucial for cancer progression.

## INTRODUCTION

Hepatocellular carcinoma (HCC) is one of the most common cancers and is the frequent cause of cancer-related death in the world [1]. Although incidence rates have been declining for most cancers, rates are increasing for HCC [2]. Despite significant improvement in both diagnostic and therapeutic modalities for cancer patients, metastasis still represents the major cause of cancer mortality [3]. It is well known that metastasis involves a series of interrelated events, including loss of the ability of cancer cells to adhere to their native tissue, invasion into the surrounding extracellular matrix, migration, and proliferation at a secondary site [4]. Nevertheless, despite the progress made in elucidating the molecular events underlying metastasis [5,6], relapse rates remain high after HCC resection, and relapse nearly always originates from metastases [7]. Our inability to combat HCC invasion and metastasis has become a major obstacle to the survival and the quality of life in HCC patients [8].

In recent years, CD147 (also called EMMPRIN), a member of the immunoglobulin superfamily, has emerged as a tumor-specific molecule. This molecule has been implicated in many aspects of tumor progression, and especially HCC metastasis [9]. In our previous studies it was shown that CD147 might promote HCC metastasis in multiple ways: by inducing matrix metalloproteinases (MMPs); by disrupting the HCC microenvironment [10]; by enhancing expression of βig-h3 [11]; and by regulating downstream metastasis-related genes, such as FAK, Girdin, Src and signal transducer and activator of transcription 3 (STAT3) [12,13]. To explore the basis of CD147 action on the atomic level, the crystal structure of its extracellular portion has been determined. The unique domain organization, overall flexibility, and diverse dimerization forms of CD147 provide structural clues about the multiple homophilic interaction-dependent functions of this molecule [14]. Furthermore, evidence strongly suggests that CD147 promotes tumor invasion by forming dimerization. Disruption of CD147 dimerization can prevent cancer metastasis effectively through attenuating MAPK activation and MMP-2 induction [15]. All of these results make CD147 an attractive drug target for preventing HCC metastasis. Indeed, a monoclonal antibody (mAb) drug targeting CD147, termed Licartin, has been proven to be a safe and beneficial treatment for HCC [16]. Nevertheless, advances in drug design and chemical synthesis have made small synthetic compounds more popular in drug screening and clinical trials [17]. Furthermore, the detailed structural information available for CD147 has allowed computational drug screening. However, to date, no small-molecule drug targeting CD147 for cancer therapy has been described.

In this study, we used an *in silico* screen to identify a novel small molecule, dubbed AC-73 (China Patent CN201310574056), as the first specific inhibitor of CD147. To validate this inhibitor's biological activities, we evaluated its effects on HCC motility, invasion and metastasis and explored the underlying molecular mechanisms. Additionally, we assessed its potential for use in HCC intervention using an *in vivo* assay.

## RESULTS

### Virtual screening and hit validation

**Figure 1:**
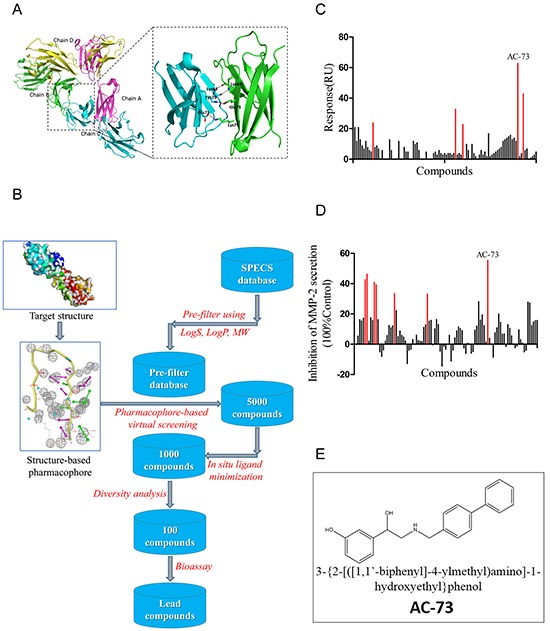
Virtual and preliminary screening of small-molecule compounds **A.** Crystal structure of CD147 (PDB: 3B5H) and enlargement of the dimerization interface. **B.** Process of virtual screening using Specs database and pharmacophore modeling. MW means molecule weight; logS and logP are two parameters representing water solubility and lipid solubility, respectively. *In situ* ligand minimization means a program in DS used for energy optimization of small molecules. **C.** The primary screen performed using the SPR assay. The binding is measured in Response Units (RU). Results showed the 100 lead compounds (black), five of them with RU > 20 (red). **D.** Results of the primary screen performed using gelatin zymography, showing the 100 lead compounds (black), seven of which had an inhibition ratio > 30% (red). The inhibition ratio (%) for MMP-2 secretion was calculated as follows: [1-gray value of MMP-2 (treatment)/gray value of MMP-2 (control)] × 100%. **E.** Chemical structure of AC-73.

The X-ray structure of CD147 (PDB: 3B5H) was used as the molecular model for our studies. Because the pockets in dimerization interface are deeply enough to bind small molecules and CD147 dimerization plays an essential role in tumor progression, as mentioned earlier, we chose the dimerization interface of CD147 to construct a pharmacophore model. The search area for screening was restricted to the C2 domain of the CD147 monomer (Figure 1A). Over 300,000 compounds from the Specs database were screened *in silico*. A total of 100 compounds were selected for biological testing after several rounds of screening (Figure 1B). Using SPR assay, the binding of 100 selected compounds to CD147 was evaluated through value of Response Units (RU) and top five compounds (RU > 20) were obtained (Figure 1C). Meanwhile, using gelatin zymography experiments, the effect of compounds on suppressing MMP-2 secretion was examined and 7 candidate compounds (inhibition ratio of > 30%) were obtained (Figure 1D). Notably, we got one compound dubbed AC-73 (Figure 1E), that presented both high RU and high effect to suppress MMP-2 secretion (Table 1).

**Table 1:** Detailed information of potential candidate compounds

Structure	IDNUMBER^a^	MW (Da)	Binding Energy (kcal/mol)^b^	SPR(RU)	Inhibition of MMP-2 secretion (% control)
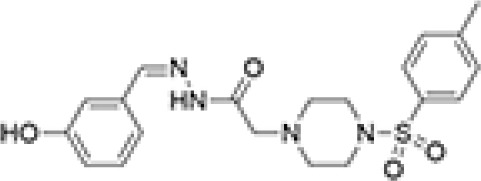	AF-399/15392135	416.493958	−69.44051	22	17.47725%
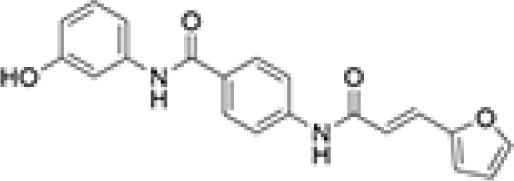	AN-979/15448127	348.352051	−61.51612	43	11.15524%
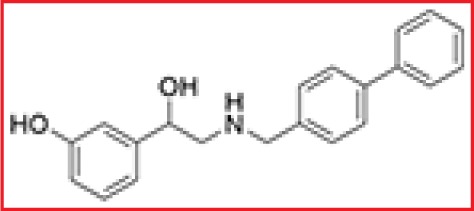	AN-465/42834501	319.36942	−72.24657	63	55.72601%
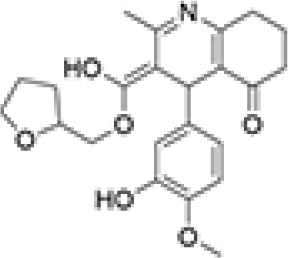	AG-205/13358154	413.463562	−125.36821	9	39.42461%
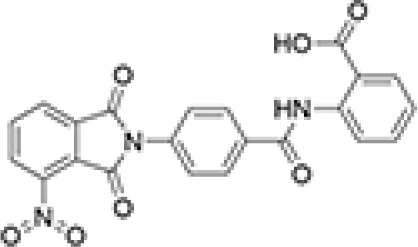	AG-205/12140154	431.354523	−89.75615	13	41.13764%

a ID number in SPECS database

b binding energy of the pose after *in situ* ligand minimization

### AC-73 inhibits CD147 dimerization

**Figure 2:**
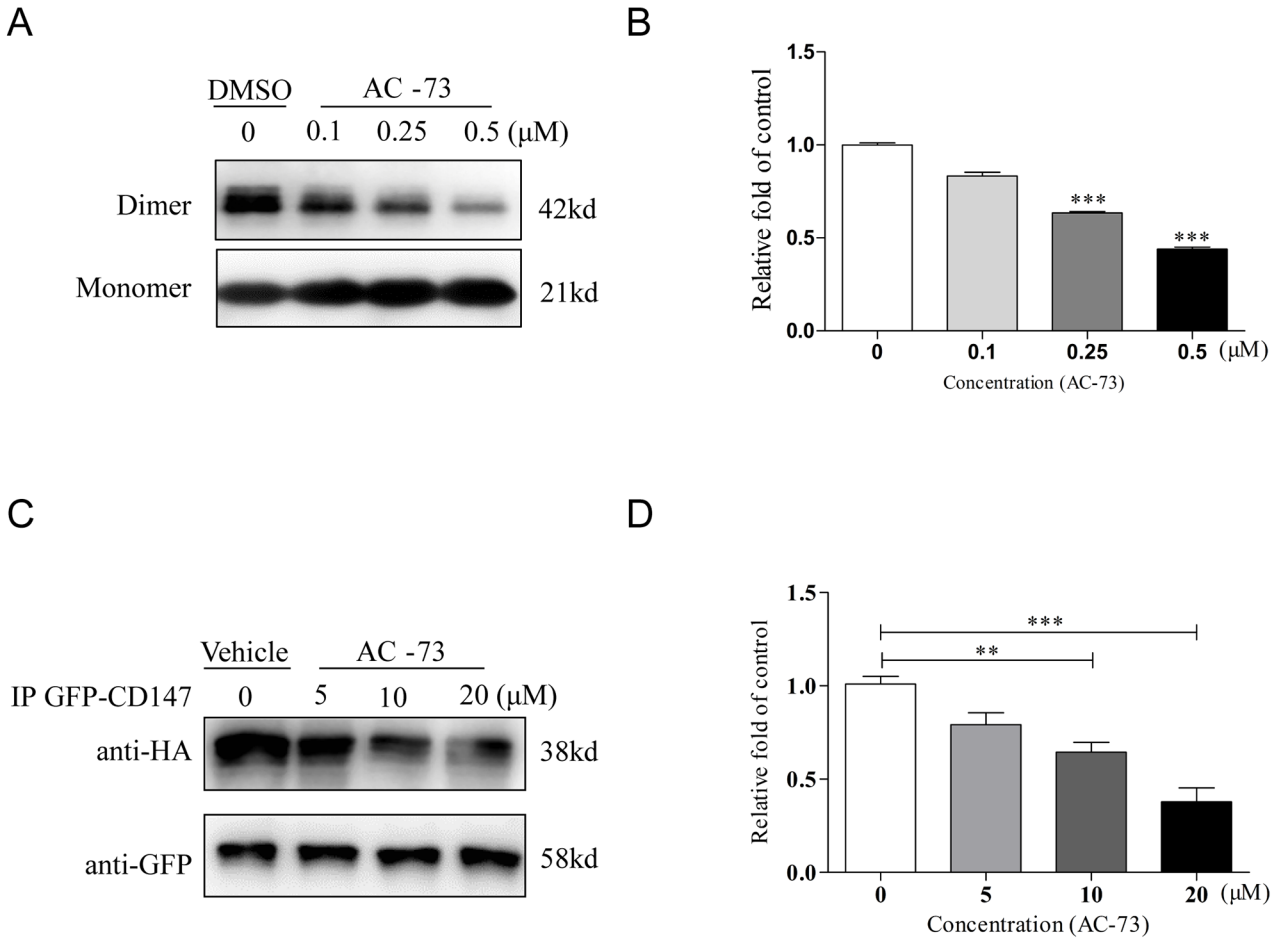
AC-73 disrupts CD147 dimerization **A.** Representative image of CD147 dimerization. A total of 5 μg of purified CD147 was mixed with 5× Laemmli sample buffer lacking SDS and different concentrations of AC-73 (0.1, 0.25, or 0.5 μM). DMSO was used as negative control. The material was then resolved on a 10% SDS-PAGE gel without boiling, followed by immunoblotting with an anti-His_6_ antibody. The dimer bands were approximately 42 kDa in size. **B.** Quantification of CD147 dimerization inhibition by densitometry analysis. **C.** AC-73 inhibited CD147 dimerization in 293T cells, as determined using a co-IP assay. **D.** Quantification of CD147 dimerization inhibition *in cellulo* by densitometry analysis. The bars represent the mean of triplicate measurements of each sample, and the error bars indicate ± SD. ***P < 0.001, **P < 0.01, *P < 0.05, one-way ANOVA (H).

Next, we verified whether AC-73 could directly disrupt CD147 dimerization. In a prokaryotic expression system, wild-type CD147 (CD147wt) was easily purified, and 5 μg of CD147wt was added to various concentrations of AC-73. The mixture was then pretreated with non-denaturing loading buffer and immunoblotted with anti-His_6_ antibody. It was observed that two major bands for CD147wt, appearing at 21 and 42 kDa, which represented the monomer and dimer of CD147 extracellular domain (CD147ECD), respectively, in solution (Figure 2A). We noticed that comparing DMSO, AC-73 could directly disrupt CD147 dimerization in a dose-dependent manner at hundreds nanomolar level (Figure 2B). To further investigate the inhibition of CD147 dimerization by AC-73 *in cellulo*, a co-immunoprecipitation (co-IP) assay was performed. HEK293T cells co-expressing CD147-HA and CD147-GFP were treated with the indicated concentrations of AC-73 for 6 hrs. Protein lysates were then purified using anti-HA magnetic beads and analyzed by Western blotting with anti-Flag and anti-GFP antibodies (Figure 2C). Strikingly, the level of CD147-GFP in the anti-Flag immunoprecipitates decreased as the concentration of AC-73 increased at the micromolar level (Figure 2D). Taken together, our results suggested AC-73 can directly inhibit CD147 dimerization both in prokaryotic expression system and in living cells.

### AC-73 decreases the motility and invasion of HCC cells by targeting CD147

**Figure 3:**
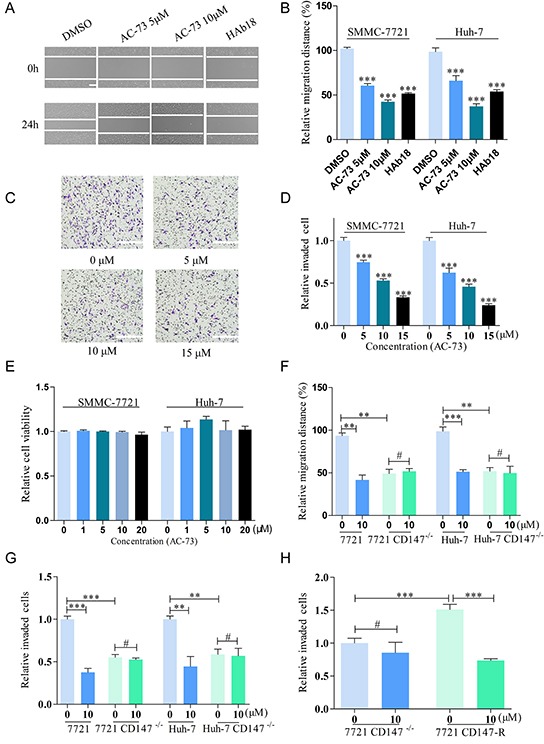
AC-73 decreases the motility and invasiveness of HCC cells via CD147 inhibition **A, B.** Effect of AC-73 on HCC cells migration. HCC cells were wounded and then incubated with varying concentrations of AC-73 (0, 5 and 10 μM) for 24 hrs. CD147 antibody HAb18 is used as a positive control. A, representative images showing SMMC-7721 cells migration, scale bars: 100 μm; B, the figure shows quantitative analysis of a wound healing assay, with triplicate measurements of three independent experiments. The relative migration distance (%) was calculated as migration distances in each drug group/ migration distances in DMSO group. **C, D.** *In vitro* invasion of HCC cells treated with AC-73 at different concentrations (5, 10, or 15 μM) for 24 hrs. C, photomicrographs illustrate representative fields of invaded cells, scale bars: 100 μm; D, The relative number of invaded cells was calculated, and the data are presented in a histogram from three independent experiments. IC_50_ was analyzed by nonlinear regression (curve fit) using GraphPad Prism V5.0 software. **E.** Effect of AC-73 on HCC cells viability. **F.** Effect of AC-73 on migration in HCC and HCC CD147^−/−^ cells. AC-73 could reduce the migration distance over 24 h for HCC cells, but not for HCC CD147^−/−^ cells. The figures show a quantitative analysis of the wound healing assay, with triplicate measurements of three independent experiments. **G.** Effect of AC-73 on the invasiveness of HCC and HCC CD147^−/−^ cells. The histogram shows a quantitative analysis of the transwell assay. **H.** Different sensitivities of SMMC-7721 CD147^−/−^ and SMMC-7721 CD147-R cells to AC-73. The quantitative analysis is presented as a histogram. All the bars represent the mean of triplicate measurements of each sample, and the error bars indicate ± SD. ***P < 0.001, **P < 0.01, *P < 0.05, ^#^*P* > 0.05, one-way ANOVA (H) for multiple comparisons (B, D and E), Student's *t*-test for two comparisons (F, G and H).

To confirm whether AC-73 could reduce the metastasis of HCC cells, we first evaluated the effect of AC-73 on the motility of HCC cells using an *in vitro* scratch assay. Treatment with AC-73 significantly decreased the migration ability of SMMC-7721 cells in a dose-dependent manner. Given that no other small molecules is known to target CD147, we used the mAb HAb18, a specific antibody against CD147 that has been described as a suppressor of the mobility of HCC, as a positive control [10]. Results showed that 10 μM AC-73 significantly inhibited approximately 50% of the migration efficacy compared with DMSO. Similar results were also obtained in Huh-7 cells (Figure 3A and 3B). Furthermore, AC-73 impaired the invasive ability of HCC cells, as assessed by a transwell assay. In Figure 3C, AC-73 decreased the invasion of two HCC cells in a dose-dependent manner at 24 hrs. In Figure 3D, IC_50_ was calculated as 10.19 μM for SMMC-7721 and 7.16 μM for Huh-7, respectively. Notably, using WST-1 assay, we also found there were no obvious effects on cell viability when two HCC cells were treated with AC-73 at a maximum concentration of 20 μM. These results implied that the inhibition of HCC cells migration and invasion by AC-73 was not due to cytotoxicity (Figure 3E).

To determine whether AC-73 affected the mobility and invasion of HCC cells by targeting CD147, we compared the responses of AC-73 in the two HCC cell lines with those in two derivative cell lines in which the CD147 gene was disrupted (Supplementary Figure S1A-S1D). All four cell lines were treated with AC-73 or DMSO for 24 hrs. Importantly, AC-73 reduced mobility in the parental HCC cell lines, but not in the CD147-deficient cells, indicating that AC-73 acted by targeting CD147 (Figure 3F). Similarly, using the transwell assay, both parental HCC cell lines showed strikingly higher sensitivity to AC-73 compared with their CD147-deficient derivatives. More specifically, 10 μM AC-73 decreased the invasive ability of SMMC-7721 and Huh-7 cells by nearly 50% but barely reduced the invasion rate in their knockout (KO) counterparts (Figure 3G).

To confirm the specific targeting, we constructed a stable cell line that harbored CD147 gene and no endogenous CD147 expressing by transfecting the plasmid pcDNA3.1-CD147 into 7721 CD147^−/−^, forming cells termed SMMC-7721 CD147-R. Notably, SMMC-7721 CD147-R showed high sensitivity to AC-73. The inhibition ratio of 10 μM AC-73 reached nearly 50% in SMMC-7721 CD147-R, compared with less than 1% in 7721 CD147^−/−^ (Figure 3H). All of these data strongly suggested that AC-73 decreased the motility and invasive ability of HCC cells by targeting CD147.

### AC-73 inhibits the invasion of HCC cells by reducing MMP-2 production through blocking CD147-stimulated MAPK/STAT3 signaling

Our previous work has shown that disruption of CD147 dimerization attenuates MAPK activation and MMP-2 induction [15]. We also noticed that the MAPK family member ERK1/2 can influence the migration and invasion of HCC cells [18] and that activity of STAT3 is necessary for invasion in HCC [19,20]. Accordingly, STAT3 knockdown reduces *in vitro* and *in vivo* invasiveness by suppressing MMP-2 in HCC [21]. A pertinent finding is that CD147 serves as an upstream activator of STAT3 signaling [22]. Furthermore, as a key transcription factor, STAT3 may be phosphorylated by ERK1/2 to drive biological functions in cancer cells [23]. Therefore, we hypothesized that AC-73 inhibited HCC metastasis by disrupting CD147 dimerization and reducing downstream ERK/STAT3/MMPs signaling.

**Figure 4:**
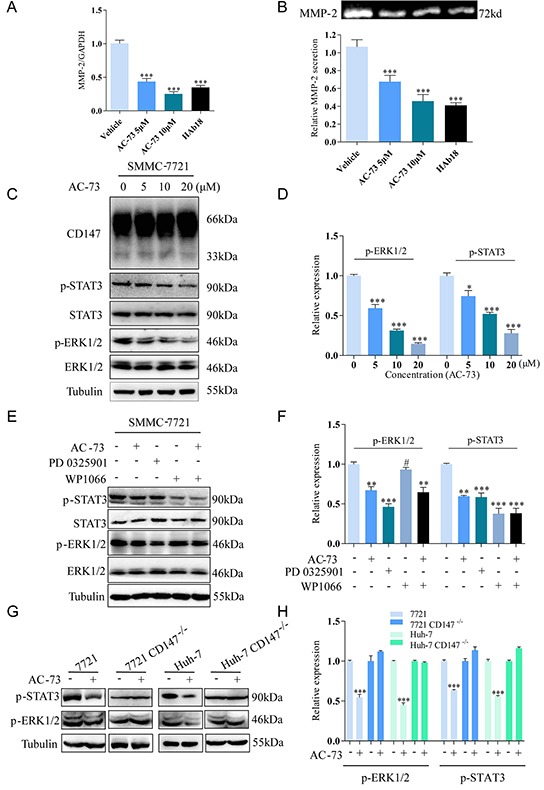
Molecular mechanism of action of AC-73 in HCC cells **A.** The effect of AC-73 on MMP-2 mRNA level in SMMC-7721 cells. MMP-2 mRNA levels were detected using real-time PCR after treatment with DMSO or different concentrations of AC-73. Here, 20 μg/ml HAb18 was used as a positive control. **B.** Gelatin zymography analysis of MMP-2 secretion. Top, representative image; bottom, quantification of the grayscale analysis in three independent experiments. **C, D.** CD147, ERK1/2 and STAT3 activities and total ERK1/2 and STAT3 expression were examined in SMMC-7721 cells treated with different concentrations of AC-73. **E, F.** ERK1/2 and STAT3 activities and total ERK1/2 and STAT3 expression were examined in SMMC-7721 cells with different treatments. AC-73 was used at a concentration of 10 μM, the ERK1/2 inhibitor PD0325901 was used at 1 μM, and the STAT3 inhibitor WP1066 was used at 10 μM. **G, H.** ERK1/2 and STAT3 activities were examined in total lysates of HCC and HCC CD147^−/−^ cells using Western blotting. All the Histograms show quantification of the gray scale analysis for western blotting. The bars represent each sample performed in triplicate and the error bars indicate ± SD. (***P < 0.001, **P < 0.01, *P < 0.05, ^#^*P* > 0.05), one-way ANOVA (H) for multiple comparisons (A, B, D and F), Student's *t*-test for two comparisons (H).

Given that MMPs are considered to be vital factors in tumor invasion and metastasis, we first evaluated whether AC-73 affected MMPs production in SMMC-7721 cells. MMPs were reported to induce tumor progression in HCC including MMP-1, MMP-2, MMP-3, MMP-7, MMP-9, MMP-11 and MMP-13 [24]. Therefore we examined the effect of AC-73 on mRNA expression of these MMPs by quantitative real-time PCR in SMMC-7721 cells. We observed AC-73 could significantly inhibit both MMP-2 and MMP-9 mRNA expression at the concentration of 10 μM, especially MMP-2, but no obvious effect on MMP-1, MMP-3, MMP-7, MMP-11 nor MMP-13 (Supplementary Figure S2A). Then further study of MMP-2 showed that AC-73 could dose dependently reduce the expression of MMP-2 mRNA level and secretion of the protein level using RT-qPCR analysis and gelatin zymography experiments, (Figure 4A and 4B). Next, we wonder whether the inhibition of metastatic behavior by AC-73 can be circumvented through overexpression of MMP-2. Both plasmids overexpressing MMP-2 (EX-Z5731-M98-5) and negative control (EX-NEG-M98) were transfected in SMMC-7721 cells respectively. After evaluation of transfection efficiency (Supplementary Figure S2B), we detected the invasion ability of SMMC-7721 cells with or without AC-73 using transwell assay. Results showed that comparing SMMC-7721 cells, overexpressing MMP-2 in SMMC-7721 cells presented stronger invasion ability. While, with the treatment of AC-73, the inhibition of metastatic behavior were circumvented comparing to the normal SMMC-7721 cells, but not totally circumvented (Supplementary Figure S2C and S2D). We thought this results may indicated that MMP-2 is the key downstream signaling molecule regulated by AC-73. However, the reduction of invasion by AC-73 were not due to only this one signaling pathway. After that, we performed western blot assay to show that AC-73 did not change the protein expression of CD147, ERK1/2 and STAT3. But the phosphorylation of ERK1/2 and STAT3 was dose-dependently suppressed in SMMC-7721 cells after treatment with AC-73 for 6 hrs. (Figure 4C and 4D). Then, we determined whether AC-73 induced phospho-STAT3 (p-STAT3) reduction via ERK1/2. Results showed p-STAT3, but not total STAT3, could be suppressed after treatment with ERK1/2 inhibitor (PD0325901). In contrast, neither phospho-ERK1/2 (p-ERK1/2) nor total ERK1/2 could be suppressed by STAT3 inhibitor (WP1066), suggesting that STAT3 acts downstream of ERK1/2 (Figure 4E and 4F). From these data, it was no obvious difference in p-STAT3 expression following treatment with WP1066 alone or with both WP1066 and AC-73, suggesting that AC-73 could specifically regulate STAT3 via ERK1/2. Notably, both inhibitors were evaluated for their roles in tumor invasion. In Supplementary Figure S2E and S2F, we found reduction of HCC cell invasion when blocked with PD0325901 and WP1066. Finally, in a parallel experiment, we explored how AC-73 affected the related signaling pathways (Figure 4G and 4H). We observed that AC-73 notably reduced p-ERK1/2 and p-STAT3 in the two parental HCC cell lines, but not the HCC CD147^−/−^ lines, indicating that AC-73 inhibits ERK/STAT3 signaling via specific binding to CD147.

Considering about both STAT3 and ERK signaling are implicated in cell growth, we also evaluated the effect of AC-73 on HCC proliferation *in vitro* and found no obvious effect when the concentrations of AC-73 is less than 20 μM within 7 days (Supplementary Figure S3A). Meanwhile, the proteins of Survivin and CylinD1, regarded as proliferation related signaling molecules, were comparable expression when treating with or without AC-73, detected by western blot. In contrary, MMP-2 expression could be inhibited in a dose-dependent manner (Supplementary Figure S3B). All these results indicated AC-73 tends to inhibit metastasis rather than proliferation in HCC.

To sum up, AC-73 inhibited HCC metastasis by disrupting CD147 dimerization and reducing downstream ERK1/2/STAT3/MMP-2 signaling.

### AC-73 reduces HCC metastasis *in vivo*

**Figure 5:**
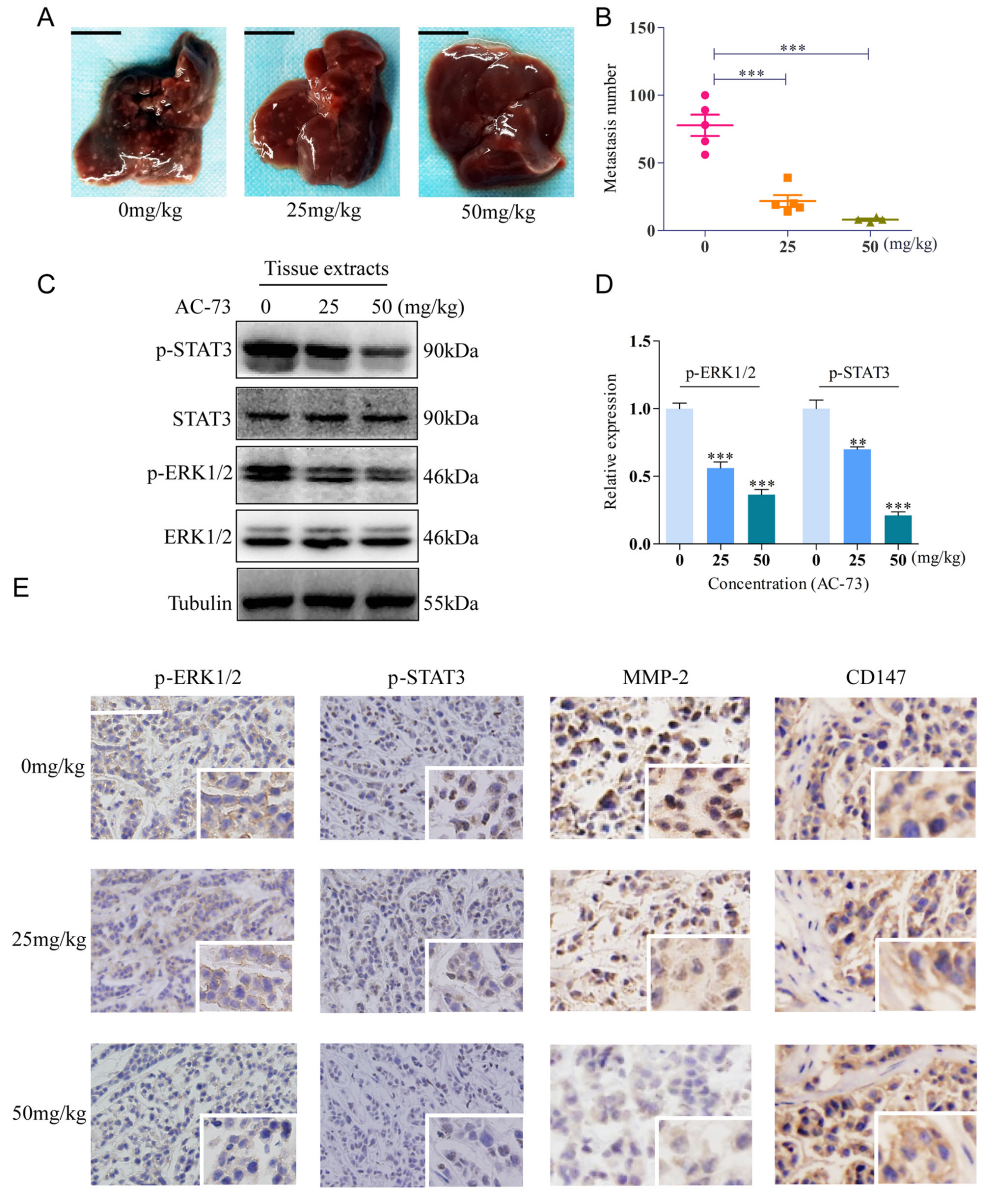
AC-73 reduced tumor metastasis by affecting the CD147/ERK1/2/STAT3 pathways *in vivo* **A, B.** Metastasis assay by intrahepatic injection of SMMC-7721 cells in nude mice. Liver were excised for examination, scale bars: 1cm. Intrahepatic metastases in each group, as determined by calculation of metastatic lesions in the whole liver (*n* = 5). **C, D.** Phosphorylation of ERK1/2 and STAT3 was examined in total lysates of original orthotopic tumors from each group, and histogram shows quantification of the gray scale analysis for western blotting. **E.** Phosphorylation of ERK1/2 and STAT3, MMP-2 and CD147 expression in original orthotopic tumors at 200× magnification, as determined by IHC staining analysis following treatment with AC-73. Each inset shows images obtained at 400× magnification, scale bars: 100 μm.

The effect of AC-73 on inhibiting HCC metastasis was assessed in an orthotopic transplant nude mouse model. We performed orthotopic implantation of SMMC-7721 cells into the left liver lobe of the nude mice. One week after implantation, when tumor is well established, the mice were treated with Cremophor EL/ethanol or different concentrations of AC-73. Enumeration of intrahepatic metastases revealed that AC-73 significantly decreased the incidence of metastatic foci in nude mice, evaluated by gross pathology (Figure 5A and 5B). Then, through tissue pathology examination of original orthotopic tumors, we found compared with Cremophor EL/ethanol, AC-73 inhibited the phosphorylation of ERK1/2 and STAT3 in a dose-dependent manner by both Western blot (Figure 5C and 5D) and immunohistochemistry (IHC) analyses (Figure 5E), suggesting that AC-73 suppresses the ERK/STAT3 pathway *in vivo*. Further, as a key downstream signaling molecule, expression of MMP-2 was detected by IHC. Results indicated MMP-2 was also reduced by AC-73. Meanwhile, we also detected CD147 expression in tissue and found no significant difference among each group (Figure 5E). Notably, all these results were as similar as we got by western blot *in vitro*. Additionally, we detected about both *in situ* and metastatic foci by histology and observed AC-73 couldn't inhibit tumor cell proliferation *in vivo* as well as *in vitro* through calculating the maximum tumor diameter of *in situ* focal (Supplementary Figure S3C and S3D).

### AC-73 presents low toxicity and well tolerance in nude mice

**Figure 6:**
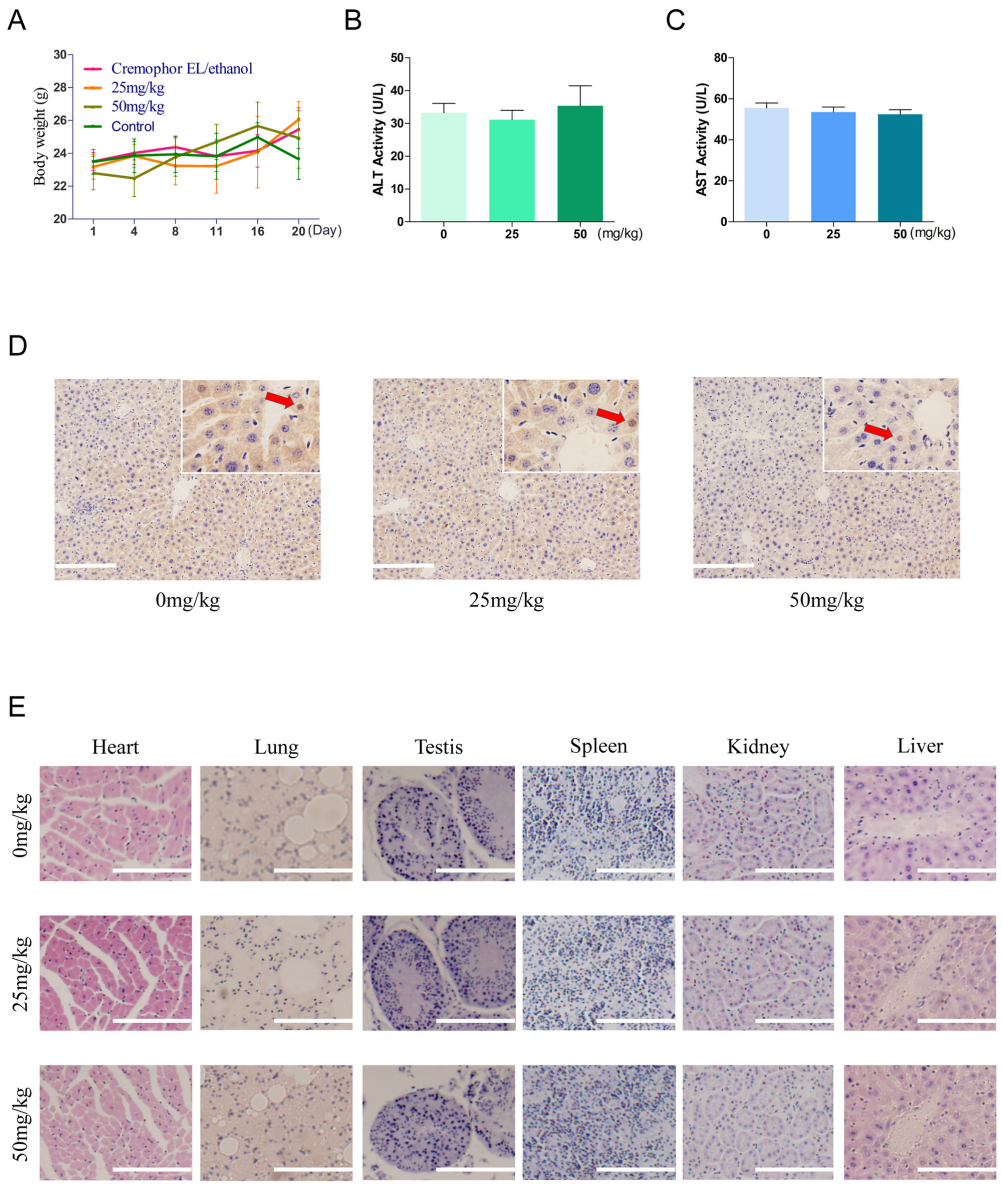
Toxicity and tolerance of AC-73 *in vivo* **A.** Effect of treatment in each experiment group on body weight. 6-week-old male nude mice were divided into 4 groups randomly (n = 5). Mice were injected with normal saline (control), Cremophor EL/ethanol (vehicle), 25 mg/kg/day and 50 mg/kg/day of AC-73. The body weight of each mouse was recorded daily. **B.** and **C.** Effect of AC-73 on ALT and AST. Mice were treated with AC-73 at the concentration of 0 mg/kg/day (Cremophor EL/ethanol), 25 mg/kg/day and 50 mg/kg/day. After 20 days, GPT/ALT and GOT/AST were measured by extracting the eyeball blood using a commercial AST or ALT assay kit. The bars represent each sample performed in triplicate and the error bars indicate ± SD. (P > 0.05), one-way ANOVA (H). **D.** Tunel staining in liver tissue at 200× magnification, following treatment with AC-73 at the concentration of 0 mg/kg/day (Cremophor EL/ethanol), 25 mg/kg/day and 50 mg/kg/day. Each inset shows images obtained at 400× magnification. Scale bars: 100 μm. Red arrows point to the positive cells (brown). **E.** Serial histologic sections of their removed hearts, lungs, testis, spleens, kidneys and livers were stained by H&E. to evaluate the toxicity of AC-73, scale bars: 100 μm.

In a toxicity test, 6-week-old male nude mice were treated with or without AC-73. Toxicity of AC-73 were evaluated by mice body weight, aspartate aminotransferase (AST) and alanine aminotransferase (ALT) catalytic activities in serum, apoptosis detection and H&E staining of major organs when mice were sacrificed after more than 20 days. Results showed that AC-73 did not cause obvious changes in body weight of nude mice in each group (Figure 6A). In addition, comparing with vehicle group (Cremophor EL/ethanol), both ALT and AST enzyme levels did not rise abnormally but basically maintained within normal range in AC-73 groups (Figure 6B and 6C). To further evaluate whether AC-73 could cause liver damage, we detected apoptosis of liver tissue in each experiment group by tunel staining and found neither vehicle nor AC-73 could significantly cause apoptosis in hepatocytes, the total number of positive cells in each group were less than 1% (Figure 6D). In the end, through H&E staining, AC-73 didn't affect other major organs comparing with vehicle group (Figure 6E). Over all, all the *in vivo* results indicated AC-73 was well tolerated and might qualify as a candidate drug for HCC Intervention.

### AC-73 likely binds to the dimer interface of CD147

**Figure 7:**
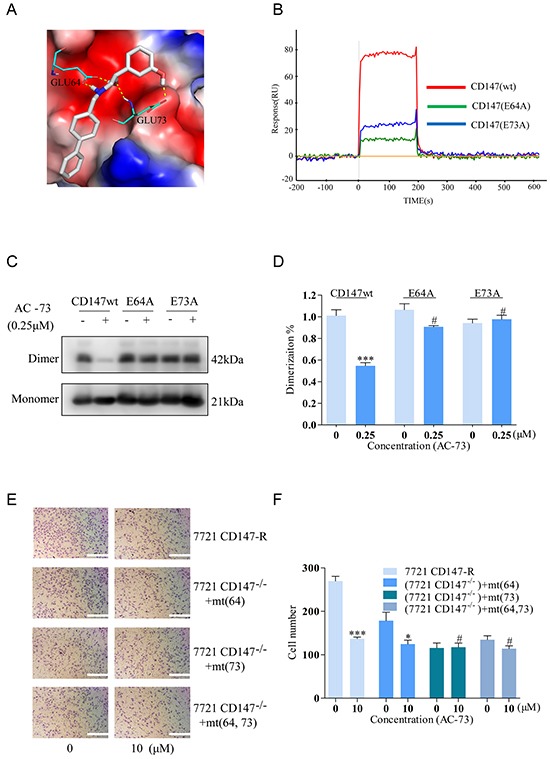
Analysis of the possible binding interface between CD147 and AC-73 **A.** Simulated average complex structure of AC-73 in the N-terminal domain of CD147. **B.** An SPR assay was performed to evaluate the response (RU) of AC-73 to the three CD147-related proteins in a Prokaryotic expression system. The red line shows a high RU, with a representative concentration of AC-73 flowing to the CD147wt protein. The blue and green lines show the low RU, with the same concentration of AC-73 flowing to the CD147mt proteins. **C.** A non-denaturing SDS-PAGE assay was performed to evaluate the dimerization of the CD147wt and CD147mt proteins following treatment with AC-73 (0.25 μM). **D.** Quantitative analysis of the non-denaturing SDS-PAGE assay, with triplicate measurements of three independent experiments. **E.** A transwell assay was performed to evaluate whether migration can be influenced by AC-73 in SMMC-7721 cells expressing mutant CD147. Three mutation plasmids—pcDNA3.1-CD147 (E64A), pcDNA3.1-CD147 (E73A) and pcDNA3.1-CD147 (E64A and E73A)—together with pcDNA3.1-CD147wt were stably transfected into SMMC-7721 cells and treated with AC-73 or vehicle. Scale bars: 100 μm. **F.** The quantitative analysis appears as a histogram. The bars represent the mean of triplicate measurements of each sample, and the error bars indicate ± SD. ***P < 0.001, *P < 0.05, ^#^*P* > 0.05, Student's *t*-test.

Having determined that AC-73 can inhibit the CD147-mediated metastatic features of HCC, we finally identified the possible key AC-73-binding residues in CD147. Using PyMOL (http://pymol.sourceforge.net/), the binding simulation (Figure 7A) revealed multiple hydrogen bonds between AC-73 and two amino acid residues (Glu64 and Glu73) in the active site. In particular, the carboxylic group of Glu64 was involved in forming two hydrogen bonds, namely, with N and the hydroxyl-group O of AC-73. Meanwhile, the side chain of Glu73 formed a hydrogen bond with the phenolic hydroxyl of AC-73. In addition, we predicted the most probable contact residues by calculating the energy contributions of those residues within 10Å of AC-73 using Glide software. As shown in Table 2, Glu64 and Glu73, located in the N-terminal domain of CD147, presented the greatest predicted energy contributions. To verify the computer simulation results, the binding of AC-73 to CD147wt and two CD147 mutants (CD147mts)—E64A and E73A—was compared by SPR assay. In contrast to the effective binding of AC-73 to CD147wt, the compound's binding to both CD147mts was negligible (Figure 7B).

**Table 2:** Per-residue interaction scores for residues within 10Å

Residue	Energy	Residue	Energy	Residue	Energy
**Lys75**	17.4635	**Phe74**	−0.5092	**Glu73**	−35.8100
**Thr72**	1.1777	**Leu67**	0.0390	**Asp65**	−13.9780
**Glu64**	−49.4590	**Lys63**	10.0810	**Leu62**	−3.8209
**Val61**	−0.5271	**Val60**	0.0789	**Trp55**	−0.3513
**Arg54**	11.3690	**His53**	−1.8172		

Energy contribution from AC-73 to every residue site of CD147 in the range of 10Å using Glide software. A greater absolute value indicates a bigger energy contribution.

To further confirm this observation, the effects of AC-73 on the dimerization of different constructs was evaluated using a native PAGE assay. As shown in Figure 7C and 7D, AC-73 could only disrupt the dimerization of CD147wt, and not that of the CD147mts. Moreover, similar results were obtained in an invasion assay of living cells expressing either CD147wt or the glutamate mutants. On the premise of almost equally CD147 expression (Supplementary Figure S1E and S1F), CD147wt rendered the cells highly sensitive to AC-73 whereas three cell lines expressing the single glutamate mutants or a double mutant (E64A and E73A) were less sensitive, suggesting that Glu64 and Glu73 of CD147 are indispensable for AC-73-dependent inhibition of CD147-mediated HCC invasion (Figure 7E and 7F). Taken together, these data demonstrated that AC-73 binds to the N-terminal IgC2 domain of CD147 predominantly through Glu64 and Glu73, although it is likely that other residues contribute.

## DISCUSSION

**Figure 8:**
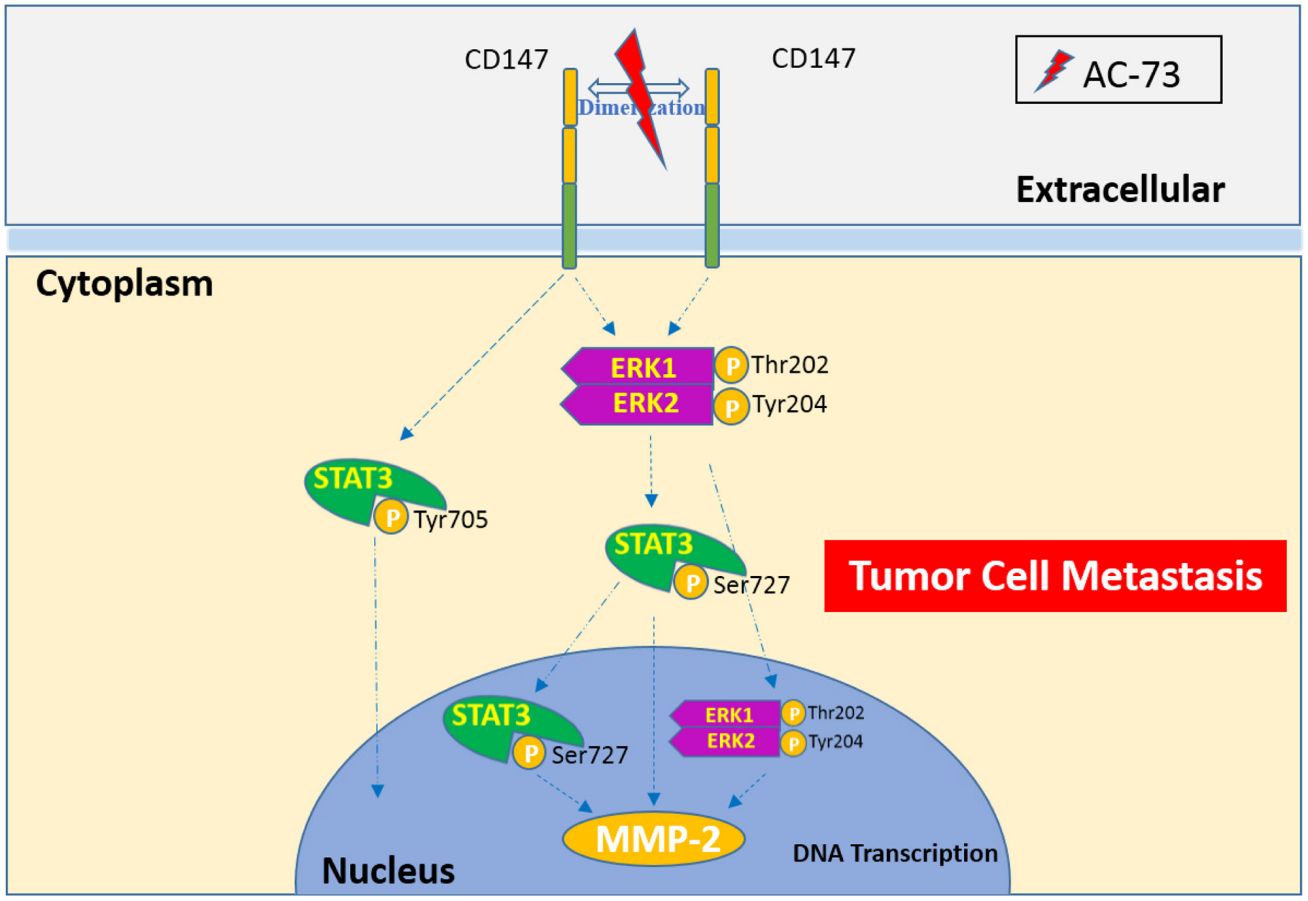
A proposed working model for AC-73-mediated suppression of CD147/ERK1/2/STAT3 signaling to inhibit HCC metastasis

In the present study, we have identified the novel candidate small-molecule compound AC-73, which binds to the N-terminal IgC2 domain of CD147, preventing CD147 dimerization. Accordingly, the reduction in CD147 signaling leads to lower MMP-2 production, apparently through suppression of the MAPK/STAT3 signaling pathway (Figure 8). The resulting diminished motility and loss of invasiveness in HCC cells appears to culminate in reduced metastasis. To our knowledge, AC-73 is the first small-molecule inhibitor targeting CD147 that may be used as a potential intervention for HCC metastasis.

In recent years, improved knowledge of the oncogenic processes and signaling pathways in HCC has revealed several potential therapeutic targets and has driven the development of molecular targeted therapies [25]. As a milestone, the tyrosine kinase inhibitor sorafenib unveiled a new direction for subsequent research[26], and many small-molecule drugs targeting specific oncogene alterations or abnormal signaling cascades are currently in clinical trials [27]. However, several of these drugs have adverse effects due to their low specificity, and most of them are designed to affect cell proliferation, differentiation and angiogenesis, rather than invasion and metastasis, which are mainly responsible for HCC mortality[7,28]. Current drugs, such as dasatinib (XL228), which targets insulin-like growth factor (IGF) signaling, and benzoic acid derivative, which blocks hepatocyte growth factor (HGF) signaling, appear to reduce HCC metastases, but their critical molecular targets remain unclear [29,30]. Thus, novel approaches to treating HCC metastasis are urgently needed.

CD147 plays important roles in cancer. Indeed, it has many characteristics of an ideal cancer target: (1) CD147 is relatively specific for cancer cells because it is not expressed or is expressed at very low levels in normal cells but is overexpressed in cancer cells [9,31–34]; (2) overexpression of CD147 is associated with malignant biological phenotypes and/or a poor prognosis [35–38]; (3) inhibition of CD147 is efficacious because it is widely implicated in motility and invasion of HCC cells [9, 16, 39–41]; (4) CD147 is very “drugable,” as a cell surface molecule that can be easily screened for small-molecule inhibition or targeted by a specific antibody [42]. Thus, targeting of CD147 may effectively delay or prevent metastasis and prolong patient survival.

There is no doubt that mAbs have become the most rapidly expanding class of pharmaceuticals for treating diverse human diseases, including cancer [43]. Given that CD147 is a potential drug target in cancer, our laboratory has developed Licartin, an ^131^I-labeled antibody against CD147 that has been approved as a new drug for the treatment of primary HCC by the China State Food and Drug Administration (No. S20050039, April 2005). In a clinical trial, detailed results showed that Licartin significantly decreased the tumor recurrence rate by 30.4% and increased the survival rate by 20.6% [44]. However, the inclusion of radioactive ^131^I in Licartin may become a potential problem for large-scale clinical application. Moreover, the possible immunogenicity of Licartin, resulting in the development of human anti-mouse antibodies (HAMA), should be prevented. In contrast, AC-73 is relatively safe, specific for its binding target and well tolerated *in vivo*. Additionally, similar to most small-molecule drugs, AC-73 has the advantages of ease of manufacture, low cost and the prospect of oral bioavailability [45–47]. Furthermore, the results showing anti-metastatic activity for AC-73 due to a reduction in MMP production are similar to those obtained with the treatment of CD147-blocking mAbs [10]. Consequently, it is a worthwhile goal to characterize small-molecule drugs targeting CD147. Notably, the anti-CD147 antibody HAb18 was included as a positive control in the present work because no other small molecules targeting CD147 have been reported thus far, and we needed to verify the efficacy of AC-73 and the accuracy of our experimental system. However, this antibody would not be the most appropriate positive control for AC-73 in most *in vitro* assays due to these inhibitors’ different natures and quantities.

In this present work, we focused on AC-73 efficacy in HCC. Actually, a variety of important risk factors for the development of HCC have not been ignored. These include chronic hepatitis B virus (HBV) infection, chronic hepatitis C virus (HCV) infection, alcohol intake and cirrhosis of almost any cause [48]. Interestingly, our previous study reported that highly expressed CD147 not only involves HCC parenchymal cells, but also exists in the perisinusoidal area of liver cirrhosis patients, suggesting this molecule may have a role in liver fibrosis and cirrhosis [49]. Therefore, as a target of CD147, AC-73 or its derivative could be supposed to reverse Cirrhosis progress effectively in the future.

Indeed, as a lead compound, AC-73 needs to be modified in further studies. First, the exact binding interface between AC-73 and CD147 should be further explored in structural experiments. Second, the unique chiral group in AC-73 should be improved to make the compound easily synthesizable and purifiable.

Finally, as we know, liver plays vital role in the pharmacokinetics of the majority of drugs. Therefore, it is necessary to study the pharmacokinetics of modified compounds based on AC-73. The related metabolic parameters of compounds should be evaluated to ensure hepatocytes and even abnormal liver cells have the ability to metabolic compounds in a certain range of doses. Taken together, AC-73 should be modified to increase the activity and ensure safety in the future.

In conclusion, our findings reveal a promising therapeutic application of AC-73. Such an approach may have benefits in reducing HCC metastasis when used either alone or in combination with other therapies.

## MATERIALS AND METHODS

### Compound

AC-73 (3-{2-[([1, 1′-biphenyl]-4-ylmethyl) amino]-1-hydroxyethyl} phenol) was screened and purchased from the Specs chemistry database (Specs ID number AN-465/42834501). In this present work, a total of two vehicles were used. The compound was dissolved in 20% DMSO (Sigma, St. Louis, MO) and diluted in DMEM to the desired concentration, with a final DMSO concentration of no more than 0.2% for all *in vitro* studies. For *in vivo* experiments, AC-73 was dissolved in Cremophor EL/ethanol (50:50; Sigma Cremophor EL, 95% ethyl alcohol) at 4-fold of the highest dose and stored at room temperature.

### Cell culture

The HCC cell lines SMMC-7721 (7721) and Huh-7 were purchased from the Institute of Cell Biology of Academia Sinica (Shanghai, China) and the Cell Bank of the JCRB (Tokyo, Japan), respectively. Two CD147-KO HCC cell lines—7721 CD147-KO (7721 CD147^−/−^) and Huh-7 CD147-KO (Huh-7 CD147^−/−^)—were constructed using the zinc-finger nuclease approach as previously reported [50] and CRISPR-Cas9 genome editing, respectively, in parallel tests. Human monocytic THP-1 cells were purchased from the American Type Culture Collection (ATCC; Manassas, VA, USA). HEK293T cells were purchased from the Cell Bank of the Type Culture Collection of the Chinese Academy of Sciences (Shanghai, China). All cells have been authenticated by short tandem repeat profiling [51]. The cells were cultured in DMEM supplemented with 10% FBS, 1% penicillin/streptomycin, and 2% L-glutamine at 37°C in a humidified atmosphere of 5% CO_2_.

### Reagents

An antibody against CD147 (mAb HAb18) was prepared as described in previous study [52]. Antibodies against α-tubulin (sc-8035) and GFP (A1514) were purchased from Santa Cruz (Dallas, TX). An antibody against HA (AH158) was purchased from BYT (China). Antibodies against His_6_ and ERK1/2 (4955S) were obtained from Cell Signaling Technology (Boston, MA). Antibodies against p-ERK1/2(Thr202/Tyr204) (ab131438) were purchased from Abcam (Cambridge, UK). Antibody against Survivin (10508-1-AP) was purchased from Proteintech Group (China). Antibody against CyclinD1 (ARE6024) was purchased from AR (China). Antibody against MMP-2 (GTX104577) was purchased from GeneTex (United States). Antibodies against STAT3 and p-STAT3 (Ser727) were obtained from Biorbyt LLC (San Francisco, CA). The STAT3 inhibitor WP1066 (Merck Millipore, Darmstadt, Germany) and the ERK1/2 inhibitor PD0325901 (BD Biosciences) were also used in the present study.

### Plasmids and site-directed mutagenesis

Three plasmids—pCMV-HA-CD147, pEGFP-N1-CD147 and pcDNA3.1-CD147 (WT)—were constructed in this present work. Briefly, the following plasmids were used: pCMV-HA (Clontech, Mountain View, CA, US), peGFP-N1 (Clontech, Mountain View, CA, US), pcDNA3.1 (Invitrogen, Carlsbad, CA, USA). The coding sequence of CD147 (NM_198589) was inserted into pCMV-HA with Hind III and Xho I, into peGFP-N1 with Xho I and BamH I, and into pcDNA3.1 with EcoR I and Xho I to produce pCMV-HA-CD147, pEGFP-N1-CD147 and pcDNA3.1-CD147 (WT) seprately. Another three mutation plasmids, termed pcDNA3.1-CD147 (E64A), pcDNA3.1-CD147 (E73A) and pcDNA3.1-CD147 (E64A and E73A), were prepared for a parallel controlled trial. Plasmids of overexpressing MMP-2 (EX-Z5731-M98-5) and negative control (EX-NEG-M98) were bought by GeneCopoeia. Site-directed mutagenesis was performed with the QuikChange Lightning Multi Site-Directed Mutagenesis Kit (Stratagene) in an expression plasmid encoding the CD147-pEGFP-N1 fusion protein. The following primer pairs were used: E64A (forward, 5′-GTGGTGCTGAAGGCGGACGCGCTGCC-3′; reverse, 5′-GGCAGCGCGTCCGCCTTCAGCACCAC-3′) and E73A (forward, 5′-GGCCAGAAAACGGCGTTCA AGGTGGAC-3′; reverse, 5′-GTCCACCTTGAACGCCGT TTTCTGGCC-3′). Double mutants were also produced according to the manufacturer's instructions. The introduction of the mutations into the cDNAs was verified by DNA sequencing and cell transfection, as previously described [53].

### Expression and purification of extracellular portion of HAb18G/CD147 tagged with 6 × His

Extracellular portion of CD147 (residues 22–205 of CD147) was inserted into pET21a (+) (Novagen) with *NdeI* and *XhoI*. The construct was transformed into the *Origami(DE3)* strain and grown in LB, yielding secretion of soluble CD147 extracellular portion by adding isopropyl-1-thio-A-D -galactopyranoside (IPTG) to a final concentration of 0.1 mM (culture for 16 h at 24°C). Harvested cells were suspended in lysis buffer (20 mM potassiumphosphate, pH 8.0, 500 mM NaCl, 1 mg/ml lysozyme (RocheApplied Science), 1mM phenylmethylsulfonyl fluoride, aprotinin, pepstatin, andleupeptin) and lysed on a French press at 20,000 p.s.i. The cell lysate was centrifuged twice at 12,000g for 45min. The soluble fraction was applied to a His-Trap chelating 5-ml column (AmershamBiosciences Co., Germany), previously charged with Ni^2+^, in an Akta-FPLC. Eluted product was further purified a Superdex 75 gel-filtration column. The antigen was identified through the molecular weight and Western blot.

### Pharmacophore model and virtual screening

A structure-based pharmacophore model was constructed to characterize the possible interactions between the dimerization area of CD147 and hit compounds. Virtual screening was performed using the Specs database, which contains more than 300,000 commercially available compounds. The detailed procedures are described as follows.

The X-ray crystal structure of HAb18G/CD147 was retrieved from the Protein Data Bank (PDB ID: 3N5H), and one monomer was extracted and prepared using Discovery Studio. Previous studies have shown that the N-terminal domain of CD147 plays an important role in its dimerization and physiological functions [15]. Thus, an active-site sphere was located in the region covering the key residues and used to create a Ludi interaction map, which consisted of a hydrogen bond acceptor and donor with hydrophobic features. Furthermore, exclusive volumes were added as constraints. This approach generated a large number of features, and thus, hierarchical clustering was performed. To this end, the pharmacophore features of each cluster were averaged, and only the center was used for further study. Next, the Screen Library protocol in Discovery Studio was used to enumerate possible subsets of pharmacophores and to screen the ligands against each pharmacophore. Each pharmacophore subset contained a minimum of 3 and a maximum of 7 features. During virtual screening, the protocol analyzed large collections of features and extracted the most relevant ones for each query ligand. According to the fitting value, the top 5,000 ligands were selected and submitted to further *in situ* ligand minimization, followed by a free energy binding calculation. Finally, the top 1,000 compounds with the best free energy binding were selected for conformational and structural diversity analysis. We chose 100 of the highest-scoring compounds from the biological test.

### Surface plasmon resonance (SPR) assay

SPR measurements were performed using the ProteOn XPR36 system with ProteOn GLH sensor chips. ProteOn PBS/Tween running buffer (phosphate-buffered saline, pH 7.4, with 0.005% Tween 20) containing 0.1% DMSO was used as a running buffer throughout, and all experiments were performed at 25°C. Purified CD147wt or CD147mt was immobilized on a GLH chip. Each compound was used at the same concentration (100 μM) and simultaneously injected in the horizontal direction, with running buffer injected as a control. Dissociation was monitored for 15 min. The data were analyzed using ProteOn Manager Software, version 2.0.

### WST-1 cell proliferation and cytotoxicity assay

To evaluate the effect of AC-73 on cell proliferation and viability, a WST-1 assay (Roche, Mannheim, Germany) was performed according to the manufacturer's instructions. Specifically, for cell viability test, SMMC-7721 and Huh-7 cells were first seeded on 96-well plates (5.0 × 10^3^ cells/well)with 100μl media and incubated at 37°C overnight. After treatment with various concentrations of AC-73 (1.0, 5.0, 10.0 or 20.0 μM) for 48 hrs. For proliferation test, 7721 cells were seeded on 96-well plates (2.0 × 10^3^ cells/well) with 100μl media containing 3 concentrations of AC-73 (0, 10.0 or 20.0 μM) and incubated at 37°C for 0.5, 1, 3, 5, 7 days, respectively. After incubation, 10 μl WST-1 reagent was added to each well, and the cells were incubated at 37°C for 2 hrs. The plates were read on a microplate reader (OD450) after being shaken thoroughly [54].

### Non-denaturing SDS-PAGE assay

To evaluate whether AC-73 could inhibit the dimerization of CD147 in a Prokaryotic expression system, non-denaturing SDS-PAGE followed by a Western blot assay was used as previously described [15]. Both CD147wt and CD147mts were purified, and 5 μg of each was added to various concentrations of AC-73 (0, 0.1, 0.25, or 0.5 μM) and mixed with 5× Laemmli sample buffer lacking SDS, followed by resolution by 10% SDS-PAGE without boiling and immunoblotting with anti-His_6_ antibody.

### Co-IP assay

This assay was performed to evaluate the effect of AC-73 on CD147 dimerization in living cells. HEK293T cells were co-transfected with pCMV-HA-CD147 and pEGFP-N1-CD147. After 24 h, cells were treated with AC-73 or vehicle control in low-FBS media (0.2% FBS) for an additional 6 hrs. Cell lysates were prepared, and 30 μg of each protein sample was used for a co-IP assay according to the manufacturer's protocol. The eluted protein samples were subjected to SDS-PAGE and Western blotting.

### *In vitro* scratch assay

An *in vitro* scratch assay was performed to evaluate the mobility of HCC cells. Cells (SMMC-7721 and Huh-7) were seeded on 24-well plates. After 24 h of culture, each well was manually scratched with a 10 μl pipette tip, washed with PBS three times and incubated with AC-73 (5 or 10 mM) at 37°C. The anti-CD147 antibody HAb18 was used as a positive control. The scratch area was photographed 24h later. The distance between two cell edges was analyzed using Image J software (NIH).

### Invasion assay

The transwell system (24 wells, 8 mm pore size, polycarbonate membrane; Corning Costar, Lowell, MA, USA) was coated with Matrigel (BD Biosciences) and used for the *in vitro* invasion assays. A total of 1 × 10^5^ cells were suspended in 300 μl serum-free media, and AC-73 (5 and 10 μM) was added to the upper chambers. Then, 500 μl DMEM containing 20% FBS was added to the lower chamber. After 24 h, the transwells were moved to a fresh 24-well plate and stained with 0.2% crystal violet for 20 min. The number of cells that had attached to the lower surface was counted in five randomly selected fields under a light microscope and statistically analyzed.

### RNA extraction, reverse transcription and real-time quantitative polymerase chain reaction (RT-qPCR)

Total RNA was extracted using the TRIzol Reagent (OMEGA Bio-Tek).

Reverse transcription was performed using the PrimeScript RT Reagent Kit (TaKaRa Biotechnology). All primers including MMP-1, MMP-2, MMP-3, MMP-7, MMP-9, MMP-11, MMP-13 and GAPDH were synthesized by Shanghai Sangon Co. (Sangon, Shanghai, China) Real-time PCR was performed using the SYBR Premix Ex Taq II Kit (TaKaRa Biotechnology).

### Gelatin zymography experiments

Gelatin zymography experiments were performed as described previously[55]. For preliminary screening, THP-1 cells were incubated with each of 100 compounds at a concentration of 50 μM. To check the secretion of MMP-2, cells were incubated with or without AC-73 at 37°C for 5 to 24 hrs. Media samples were then centrifuged to remove cellular debris, and the supernatant was collected. To evaluate the effect of AC-73 on MMP-2 secretion in HCC cells, SMMC-7721 cells were cultured with or without AC-73 in serum-free media and incubated at 37°C for 5 to 20 hrs. After which the supernatant was obtained. Each sample suspension (30μl) was mixed with SDS sample buffer without reducing agent and loaded onto a 10% polyacrylamide gel containing 0.1% gelatin. After electrophoresis, the gels were washed twice in 2.5% Triton X-100 for 15 min and then incubated in incubation buffer for 16 hrs. Next, the gels were stained with Coomassie brilliant blue and destained. The inhibition ratio (%) for MMP-2 secretion was calculated as follows: (1-gray value of MMP-2 in the treatment group/gray value of MMP-2 in the control group) × 100%.

### Western blot analysis

HCC cells were suspended in serum-free media on a six-well plate at a density of 2.5 × 10^5^/ml. A total of 2 ml of cell suspension was utilized, containing different concentrations of drug or inhibitor. After cell attachment, the conditioned media were collected. Tumor samples were minced on ice in prechilled lysis buffer containing phenylmethylsulfonyl fluoride, protease inhibitors, and phosphatase inhibitors (KeyGen BioTech, China). The homogenized tissues and cell lysates were then centrifuged at 14,000 rpm at 4°C for 15 min. The BCA Protein Assay Kit (Pierce Biotechnology, Rockford, IL) was employed to determine the total protein density and to ensure that equal amounts of proteins were separated on the 10% SDS-PAGE gel and transferred to a polyvinylidene fluoride (PVDF) membrane (Millipore, Boston, MA). After blocking with 5% nonfat milk for 1 hr. The membrane was incubated with the designated primary antibody at 4°C overnight. Immunodetection was performed using the Western-Light chemiluminescent detection system (Applied Biosystems, Foster City, CA) after incubation with the secondary antibody for 1 hr.

### Establishment of the orthotopic transplant nude mouse model of HCC metastasis

Male BALB/c nu/nu mice, 4 to 6 weeks of age, were provided by the Laboratory Animal Research Center of FMMU, and the animal study was reviewed and approved by the FMMU Animal Care and Use Committee. The mice were housed in a standard animal laboratory under constant environmental conditions, including a 12-h light and dark cycle, with free access to water and food. A mixture of SMMC-7721 cells (1 × 10^6^) in 0.1 ml culture medium and the same volume of diluted Matrigel was injected into the left liver lobe of the nude mice. The treatment was started 1 week after implantation. The mice were divided into three groups: vehicle control (Cremophor EL/ethanol), AC-73 (25 mg/kg/day) and AC-73 (50 mg/kg/day). The mice were sacrificed 4 weeks after implantation. The number of intrahepatic metastases was calculated and statistically analyzed. Tumor tissues were then fixed, embedded in paraffin, and serially sectioned at a thickness of 4 mm. IHC staining was performed, and the sections were examined by a pathologist to verify the presence of tumors.

### Toxicity test

6-week-old male nude mice were divided into 4 groups randomly (*n* = 5). Mice were injected with normal saline (control), Cremophor EL/ethanol (vehicle), 25 mg/kg/day and 50 mg/kg/day of AC-73. The body weight of each mouse was recorded daily. After 20 more days, mice were killed and selected tissues were fixed in4% paraformaldehyde. Serial histologic sections of their removed hearts, lungs, testis, spleens, kidneys and livers were stained by Hematoxylin and eosin (H&E). The concentration of the serum GPT/ALT and GOT/AST were measured by extracting the eyeball blood using a commercial AST or ALT assay kit (Nanjing Jiancheng Bioengineering Institute). Liver tissue apoptosis were detected by tunel stain (Tunel stain kit from KeyGEN BioTECH, China).

### Statistical analysis

All experiments were performed in triplicate, and the results are expressed as the mean ± SD. Statistics were evaluated using GraphPad Prism V5.0 software (GraphPad Software, La Jolla, CA). The statistical analysis was carried out using one-way ANOVA (multiple comparisons) and Student's *t*-test (two comparisons, or two tailed). Differences were deemed significant if P < 0.05. *** indicates *P* < 0.001, ** indicates *P* < 0.01, * indicates *P* < 0.05, and ^#^ indicates *P* > 0.05. IC_50_ was analyzed by nonlinear regression (curve fit) in the GraphPad Prism 5.0 software.

## SUPPLEMENTARY FIGURES


