# Supplementary material for: A novel small-molecule compound targeting CD147 inhibits the motility and invasion of hepatocellular carcinoma cells
Source: Oncotarget. 2016 Jan 23;7(8):9429–47. doi: 10.18632/oncotarget.6990 (PMC4891050; doi:10.18632/oncotarget.6990)
Supplement: Supplementary file 1 [file oncotarget-07-9429-s001.pdf]

SUPPLEMENTARY FIGURES

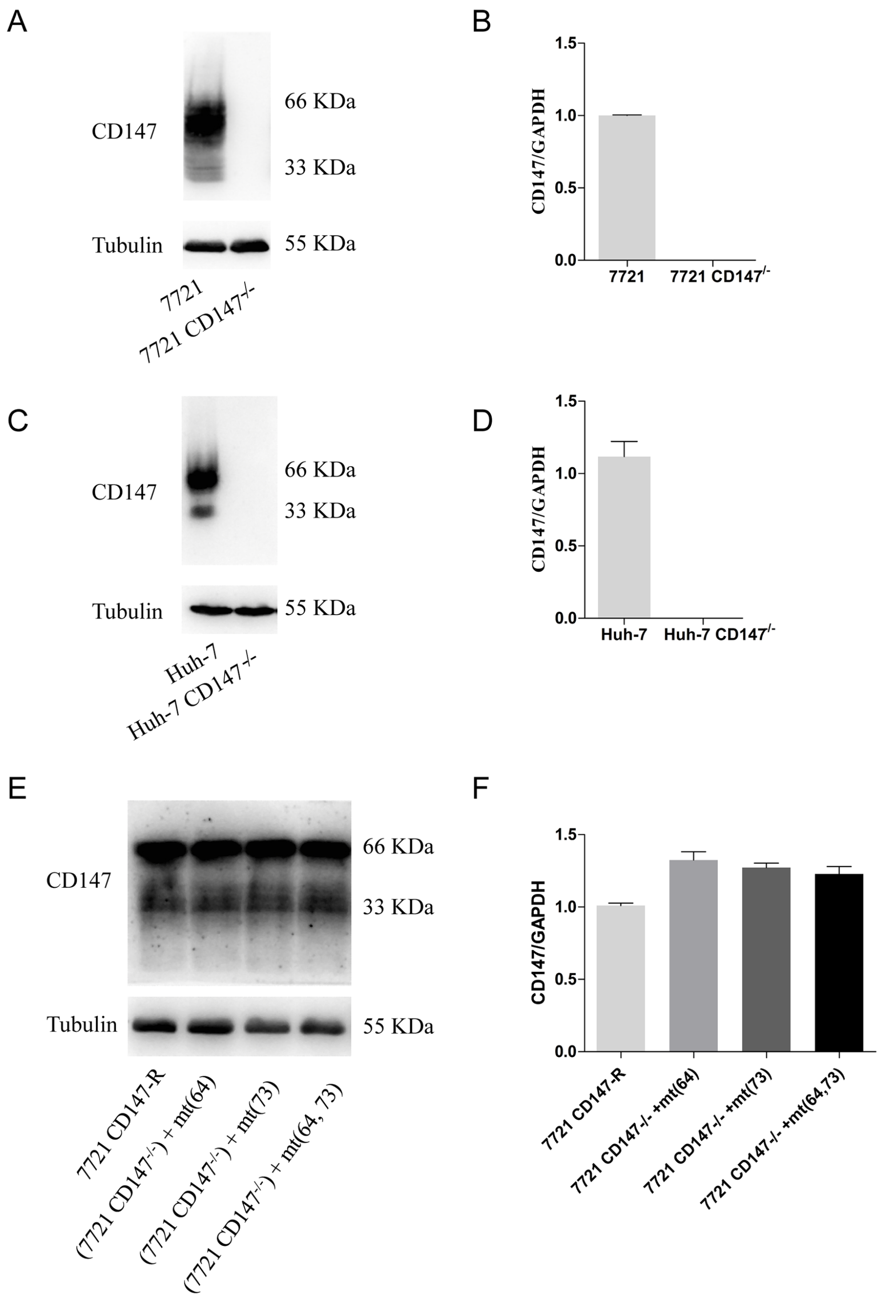

Supplementary Figure S1: Proofs of CD147 expression in constructed cell lines. A. Protein level of CD147 was examined in total lysates of SMMC-7721 cells and 7721 CD147$^{-/-}$ cells using western blotting. **B.** mRNA level of CD147 was detected in SMMC-7721 cells and SMMC-7721 CD147$^{-/-}$ cells using RT-qPCR. **C.** Protein level of CD147 was examined in total lysates of Huh-7 cells and Huh-7 CD147$^{-/-}$ cells using western blotting. **D.** mRNA level of CD147 was detected in Huh-7 cells and Huh-7 CD147$^{-/-}$ cells using RT-qPCR. **E.** Protein level of CD147 was examined in total lysates of SMMC-7721 CD147-R cells and SMMC- 7721 CD147 mutants cells using western blotting. **F.** mRNA level of CD147 was detected in SMMC-7721 CD147-R cells and SMMC-7721 CD147 mutants cells using RT-qPCR.

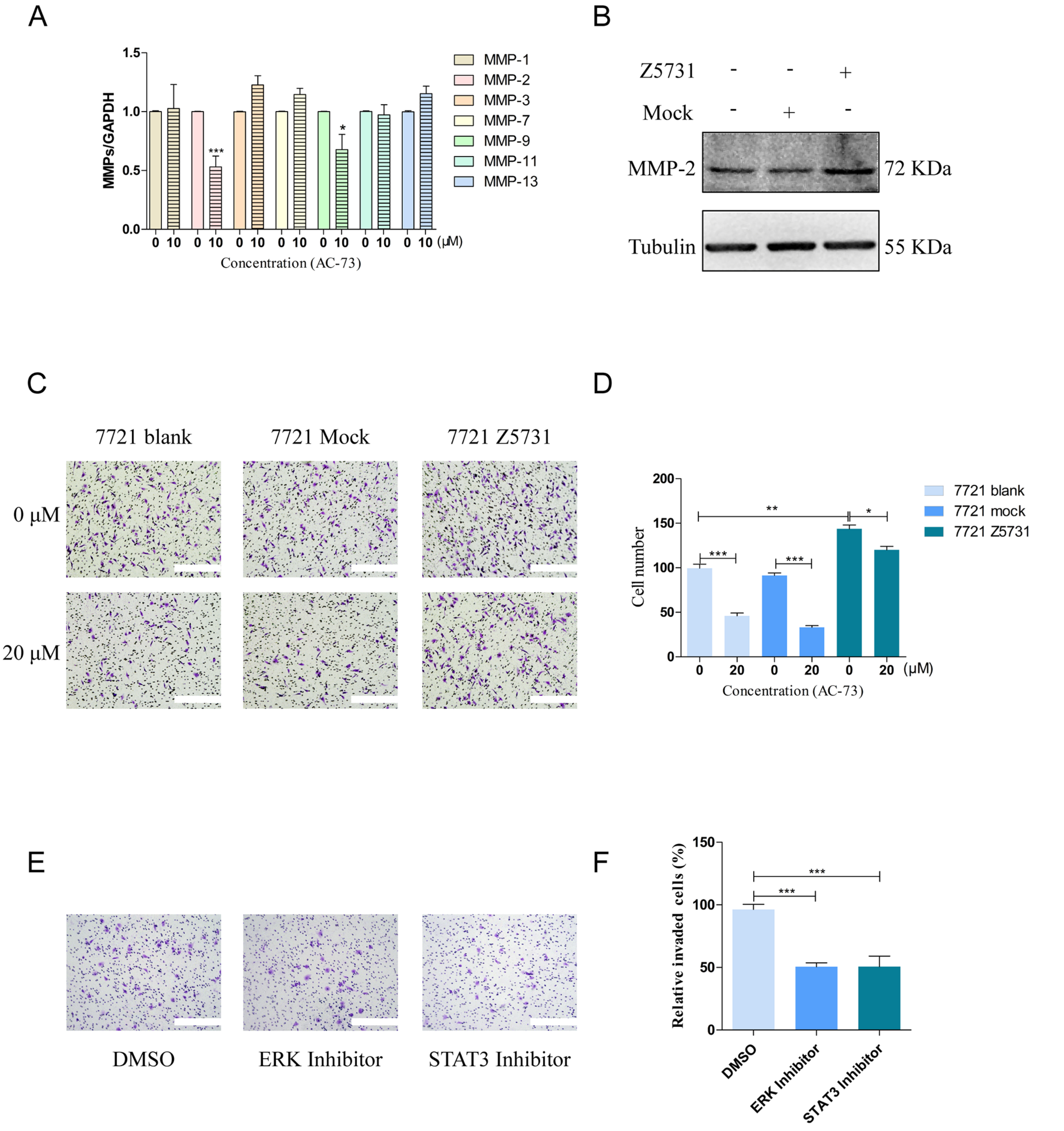

Supplementary Figure S2: AC-73 reduced tumor metastasis by affecting the CD147/ERK1/2/STAT3/MMP-2 pathways. A. MMPs (including MMP-1, MMP-2, MMP-3, MMP-7, MMP-9, MMP-11 and MMP-13) mRNA levels were detected using RT-qPCR after treatment with or without AC-73 (10 μM). **B.** MMP-2 level was assessed using western blotting in EX-Z5731-M98–5 (Z5731) or EX-NEG-M98 (Mock) transfected SMMC-7721 cells. **C.** *In vitro* invasion of three different cell lines (7721 blank, 7721 Mock and 7721 Z5731) treated with or without AC-73 (20 μM) for 24 hrs. Photomicrographs illustrate representative fields of invaded cells, scale bars: 100 μm. **D.** The relative number of invaded cells was calculated, and the data are presented in a histogram from three independent experiments. **E.** *In vitro* invasion of SMMC-7721 cells treated with ERK inhibitor (PD0325901, 1 μM) and STAT3 inhibitor (WP1066, 10 μM). Photomicrographs illustrate representative fields of invaded cells, scale bars: 100 μm. All the bars represent the mean of triplicate measurements of each sample, and the error bars indicate ± SD. ***$P < 0.001$, **$P < 0.01$, *$P < 0.05$, Student's *t*-test. **F.** The relative invaded cells was calculated, and the data are presented in a histogram from three independent experiments." should be added after "scale bars: 100 μm.

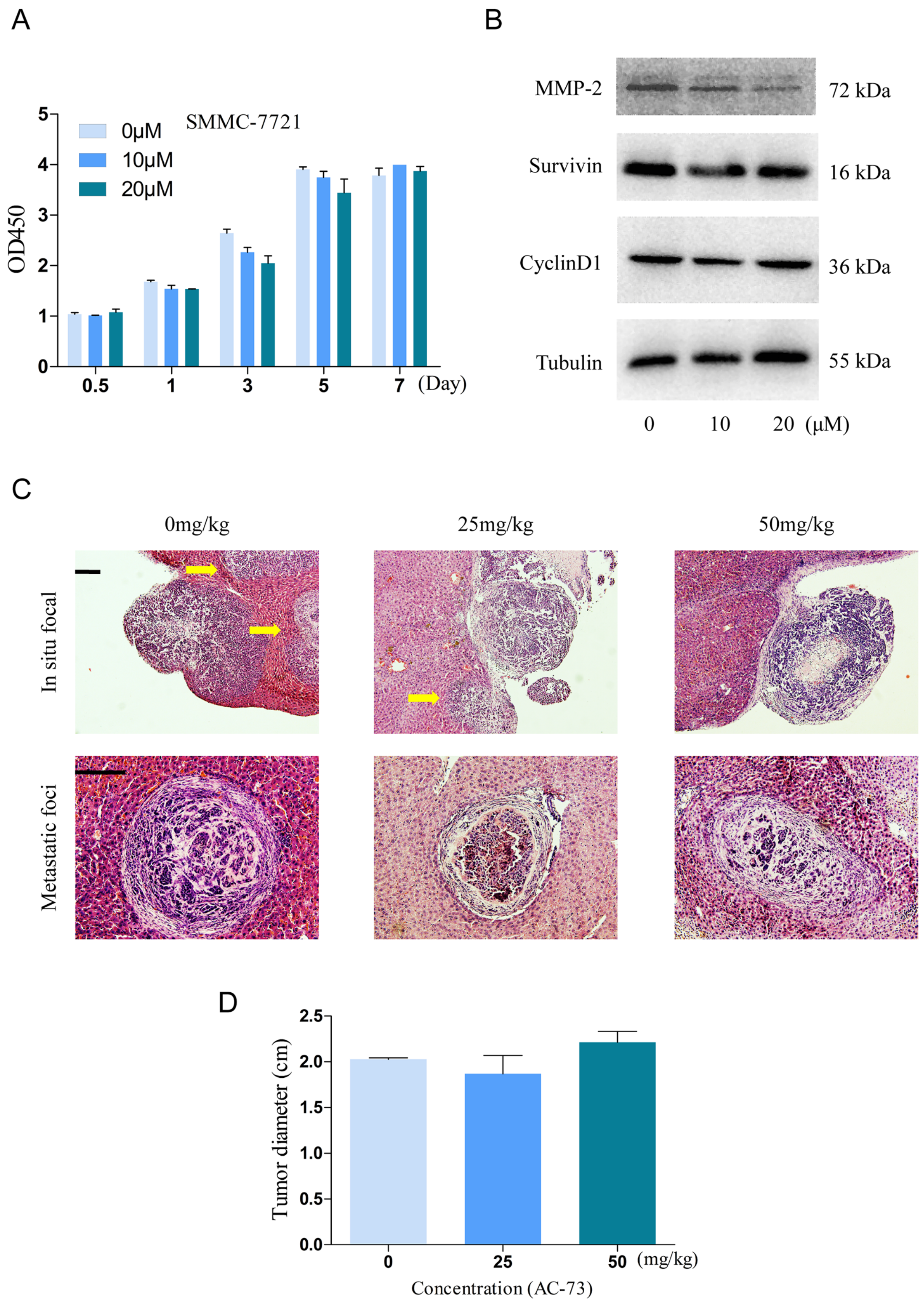

Supplementary Figure S3: Effect of AC-73 on HCC proliferation. A. Effect of AC-73 on proliferation of SMMC-7721 cells within 7 days. **B.** MMP-2, CyclinD1 and Survivin expression were examined in SMMC-7721 cells treated with different concentrations of AC-73. **C.** Representative image of in situ foci and metastatic foci by H&E staining. Upper: in situ focal were located in the edge of liver; below: metastases were surrounded by relative normal liver tissue. Both of them have clear complete capsules. Arrows indicated the metastatic sites, scale bars: 30 μm. **D.** Effect of AC-73 on proliferation in the orthotopic implantation model. Tumorigenecity was evaluated from the maximum tumor diameter. The bars represent the mean of triplicate measurements of each sample, and the error bars indicate ± SD.
